# Development of an ID-LC–MS/MS method using targeted proteomics for quantifying cardiac troponin I in human serum

**DOI:** 10.1186/s12014-023-09430-z

**Published:** 2023-09-27

**Authors:** Meltem Asicioglu, Merve Oztug, Nevin Gul Karaguler

**Affiliations:** 1 TUBITAK National Metrology Institute (TUBITAK UME), Gebze, 41400 Kocaeli, Turkey; 2 Department of Molecular Biology and Genetics, Faculty of Science and Letters, Istanbul Technical University, Istanbul, Turkey; 3 Dr. Orhan Ocalgiray Molecular Biology-Biotechnology and Genetics Research Center, Istanbul Technical University, Istanbul, Turkey

**Keywords:** Cardiac troponin I, Isotope dilution liquid chromatography tandem mass spectrometry, Reference measurement procedure, Traceability

## Abstract

**Background:**

Cardiac troponin is a complex protein consisting of the three subunits I, T and C located in heart muscle cells. When the heart muscle is damaged, it is released into the blood and can be detected. Cardiac troponin I (cTnI) is considered the most reliable and widely accepted test for detecting and confirming acute myocardial infarction. However, there is no current standardization between the commercial assays for cTnI quantification. Our work aims to create a measurement procedure that is traceable to the International System of Units for accurately measuring cardiac cTnI levels in serum samples from patients.

**Methods:**

The workflow begins with immobilizing anti-cTnI antibodies onto magnetic nanoparticles to form complexes. These complexes are used to isolate cTnI from serum. Next, trypsin is used to enzymatically digest the isolated cTnI. Finally, the measurement of multiple cTnI peptides is done simultaneously using isotope dilution liquid chromatography–tandem mass spectrometry (ID-LC–MS/MS).

**Results:**

The maximum antibody immobilization was achieved by combining 1 mg of nanoparticles with 100 μg of antibody, resulting in an average of 59.2 ± 5.7 μg/mg of immobilized antibody. Subsequently, the anti-cTnI-magnetic nanoparticle complex was utilized to develop and validate a method for quantifying cTnI in human serum using ID-LC–MS/MS and a protein calibration approach. The analytical method was assessed regarding linearity and recovery. The developed method enables the quantification of cTnI from 0.7 to 24 μg/L (R > 0.996). The limit of quantification was 1.8 μg/L and the limit of detection was 0.6 μg/L. Intermediate precision was ≤ 9.6% and repeatability was 2.0–8.7% for all quality control materials. The accuracy of the analyzed quality control materials was between 90 and 110%. Total measurement uncertainties for target value assignment (n = 6) were found to be ≤ 12.5% for all levels.

**Conclusions:**

The analytical method demonstrated high analytical performance in accurately quantifying cardiac troponin I levels in human serum. The proposed analytical method has the potential to facilitate the harmonization of cTnI results between clinical laboratories, assign target values to secondary certified reference materials and support reliable measurement of cTnI.

**Graphical Abstract:**

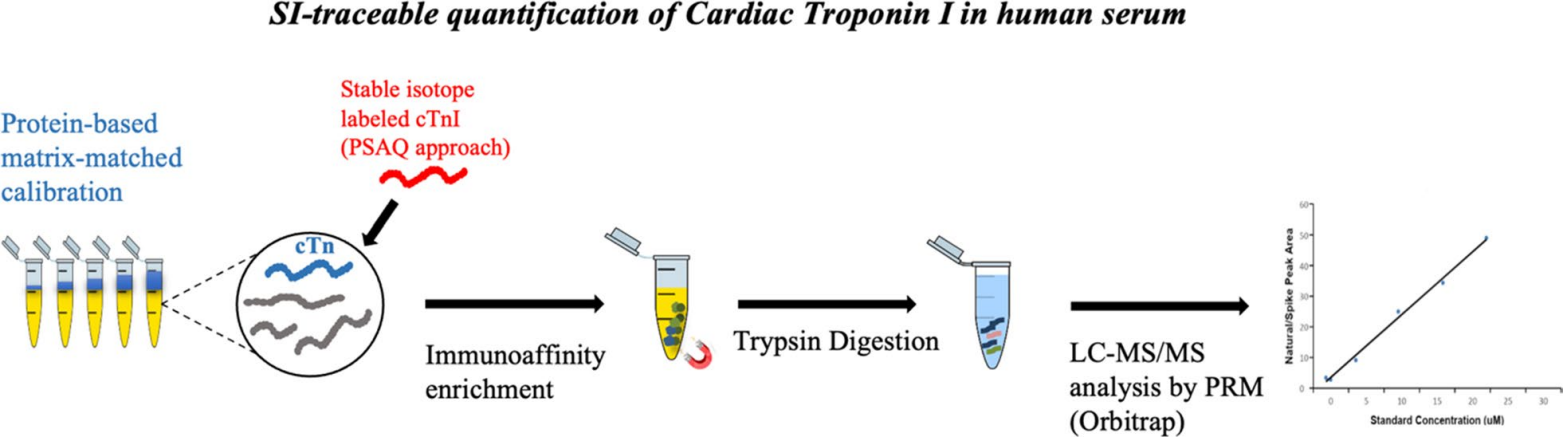

## Introduction

Cardiac troponin I (cTnI) is a protein with 110 amino acid residues. It is a widely used biomarker for the diagnosis of acute myocardial infarction [1]. The 99th percentile upper reference limit for cardiac troponin I (cTnI) in healthy individuals is 45 ng/L and following cardiac injury, cTnI is released into the bloodstream at a low abundance, typically measuring less than 50 ng/mL [2, 3]. Reliable quantification of cTnI can help reduce the risk of inaccurate diagnosis, patient mortality and healthcare costs which are currently estimated to cost the EU economy by € 210 billion a year according to the European Cardiovascular Disease Statistics (2017 edition) [4]. Variability of results, various versions of the cTnI antigens and different clinical decision cut-offs in commercial assays makes standardization difficult [5, 6]. High order RMPs and/or certified reference materials (CRM) are critical steps to improve accuracy and comparability of results and provide higher metrological order [7]. For cTnI, there is not yet a higher-level reference measurement system. Standardization and/or harmonization of cTnI assays is considered a high priority by the International Consortium for Harmonization of Clinical Laboratory Results [8]. Although the complete resolution of the standardization issue may seem challenging, the establishment of an International System of Units (SI) is a logical and necessary step to align the results of various test kits. Moreover, the standard EN ISO 17511:2020 requires the reference measurement systems, including RMPs, for the determination of analytes in human-origin samples. Having such RMP provides traceability to the SI for cTnI results which is needed by ISO 17511:2020 [9].

Isotope dilution mass spectrometry (ID-MS) is an analytical method for absolute quantification of proteins and is being developed for the SI-traceable measurement of clinically relevant proteins [10–12]. This quantitative methodology uses multiple reaction monitoring or parallel reaction monitoring (PRM) depending on the instrumentation to quantify specific protein-derived tryptic peptides and is not affected by biases like molar absorptivity or instrument variability [13]. Peptides are measured against an added internal standard to determine protein concentration. Two different strategies can be used to quantify proteins using the ID-MS methodology; the peptide-based strategy and protein standard absolute quantification [14].

In the peptide-based strategy, the internal standard is synthetic, stable isotope labeled (SIL) peptides, whereas in the protein standard absolute quantification, the internal standard is the isotope-labeled protein analogous to the target protein. The main advantage of using SIL protein as an internal standard is that the method eliminates errors and variability during sample preparation steps such as incomplete proteolysis or any material loss during sample preparation steps [15, 16].

Several studies have reported the quantification of cTnI protein by LC–MS. Kuhn et al. reported the quantification of cTnI in digested human plasma using a peptide immunoaffinity enrichment strategy coupled to the SIL-peptide [15]. Absolute quantification of cTnI from human myocardium using SIL-cTnI was demonstrated based on the ratios results from various signature peptides and their selected reaction monitoring transitions. Picard et al. have successfully demonstrated the absolute quantification of cTnI in human myocardium using SIL-cTnI. This quantification method relied on analyzing the ratios derived from various signature peptides and their selected reaction monitoring transitions [16]. Huillet et al. demonstrated the quantification of cTnI on patient serum samples using combination of immunodepleted serum samples and SDS-PAGE [17]. Zhao et al. demonstrated the quantification of cTnI in patient plasma using albumin depletion coupled with an immunoaffinity enrichment strategy [18]. Keshishian et al. demonstrated the use of a cation-exchange chromatography strategy on depleted serum to quantify a cTnI peptide using a SIL-peptide [19, 20]. Zhang et al. reported a sensitive, high-throughput, and robust Trapping-Micro-LC–MS strategy for the quantification of cTnI in swine plasma using antibody enrichment coupled with solid phase extraction (SPE) enrichment [21]. Unfortunately, the limit of detection (LOD) for MS-based cTnI assays was well above the LOD of existing commercial immunoassays and cTnI could not be detected in patient samples with the developed methods [15, 22]. Recently, a potential reference measurement method for the measurement of cTnI has been published [23]. In 2018, Schneck et al. developed the isotope dilution liquid chromatography-tandem mass spectrometry (ID-LC–MS/MS) method for measurement of cTnI in human plasma samples using an immunoaffinity enrichment approach. In this study, the researchers were able to quantify cTnI concentrations down to 2 μg/L [23]. However, this methodology has not been validated and the beads used are not commercially available.

We have developed and validated an analytical method for the accurate quantification of cTnI in human serum using ID-LC–MS/MS. To ensure traceability to the SI at clinically significant concentrations, this method utilized a protein calibrant, Standard Reference Material (SRM) 2921, and a SIL recombinant protein as an internal standard. The analytical method presented in this study, has the potential to be utilized for the standardization and evaluation of routine tests. The methodology is thoroughly described, encompassing all technical details and the calculation of measurement uncertainty, facilitating the transferability of the methods to another laboratory. The calculation of measurement uncertainty follows the guidelines outlined in the EURACHEM/CITAC Guide CG 4 (third edition) titled “Quantifying Uncertainty in Analytical Measurement” [24, 25] and is also described in detail.

## Methods

### Chemicals and materials

Human Cardiac Troponin Complex (cTn) NIST SRM 2921 was purchased from the National Institute of Standards and Technology (NIST) and used as a calibrant [26]. The cTnI-free human serum was obtained from Hytest Ltd. (Turko, Finland). SIL-protein, a monoclonal antibody against human cTnI (19c7) was obtained from Promise Proteomics (Grenoble, France). The SIL version of the monoclonal antibody’s peptide, SIL-DLPSPIER (> 95% purity 13C6, 15N4) was purchased from New England Peptide Inc (Gardner, MA, USA) as powder. Magnetic dextran nanoparticles (nanomag®-D), which particle diameters of 130 nm and the surface functionalities COOH for the covalent binding of the antibody, were obtained from Micromod Partikeltechnologie GmbH (Rostock, Germany). 1-Ethyl-3-(-3-dimethylaminopropyl) carbodiimide hydrochloride) (EDC), *N*-hydroxysuccinimide (NHS) and Pierce™ Trypsin/Lys-C Protease Mix, MS Grade were purchased from Thermo Scientific™ (Rockford, Illinois, USA). Phosphate Buffered Saline (PBS), Sodium azide and 2-(*N*-Morpholino) ethanesulfonic acid, 4-Morpholineethanesulfonic acid (MES), dithiothreitol (DTT) were purchased from Sigma-Aldrich (Darmstadt, Germany). FASP Protein Digestion Kit was obtained from Abcam (Boston, MA). Tris (Base) was purchased from Bioshop (Canada).

### Preparation of solutions, calibrators and quality control (QC) samples

All solutions and standards were prepared gravimetrically using calibrated precision balances, and the concentrations are given in mg/g. NIST SRM 2921 was used as the calibrator stock solution and was handled according to the manufacturer’s instructions.

To maintain the stability of the cTn complex over time, the calibrator working solution was prepared by diluting the calibrator stock solution to approximately 1 µg/L in a protective protein, 0.1% Bovine Serum Albumin (BSA) in Phosphate Buffered Saline (PBS). Final matrix-based calibrator levels ranging from 0.7 to 24 µg/L were prepared by diluting the calibrator working solution in 0.1% BSA in PBS. Three levels of matrix-based QC materials were prepared in a similar manner, with final concentration levels of 2.0, 5.0, and 12.0 µg/L, respectively. The SIL-cTnI protein was supplied in lyophilized form. SIL-cTnI was dissolved in 20 mM Tris buffer (containing 0.5 mM DTT) to 200 µg/L and this solution referred to as the SIL stock solution. The SIL-DLPSPIER peptide working solution was prepared in 0.1 M HCl at a concentration of 0.5 mg/g.

### Preparation of anti-cTnI-magnetic nanoparticle complex

EDC and NHS were prepared in 25 mM MES and 0.01% Tween 20 (pH:6.0). Magnetic nanoparticles (130 nm diameters) were treated with NHS and cold EDC to activate carboxyl groups and incubated at room temperature 1 h with continuous mixing. 250 µg of anti-cTnI antibody was added to the 2.5 mg (250 µL) of activated beads and incubated overnight at room temperature with continuous mixing. After immobilization, the anti-cTnI-magnetic nanoparticle complex was treated with 100 mM Tris–HCl (pH: 7.4) for 30 min with continuous mixing and placed in a magnetic separator to separate the beads. The supernatant was removed and 10 mM PBS, 0.01% Tween 20, 0.05% Sodium azide (pH: 7.4) was added to wash the beads. The magnetic beads were re-suspended using a sonicator and a wash step was performed three times. In the final step, 10 mM PBS, 0.01% Tween 20, 0.05% Sodium azide (pH: 7.4) was added to the magnetic beads and resuspended by probe sonication.

### Sample preparation and cTnI enrichment procedure using magnetic beads

**Fig. 1:**
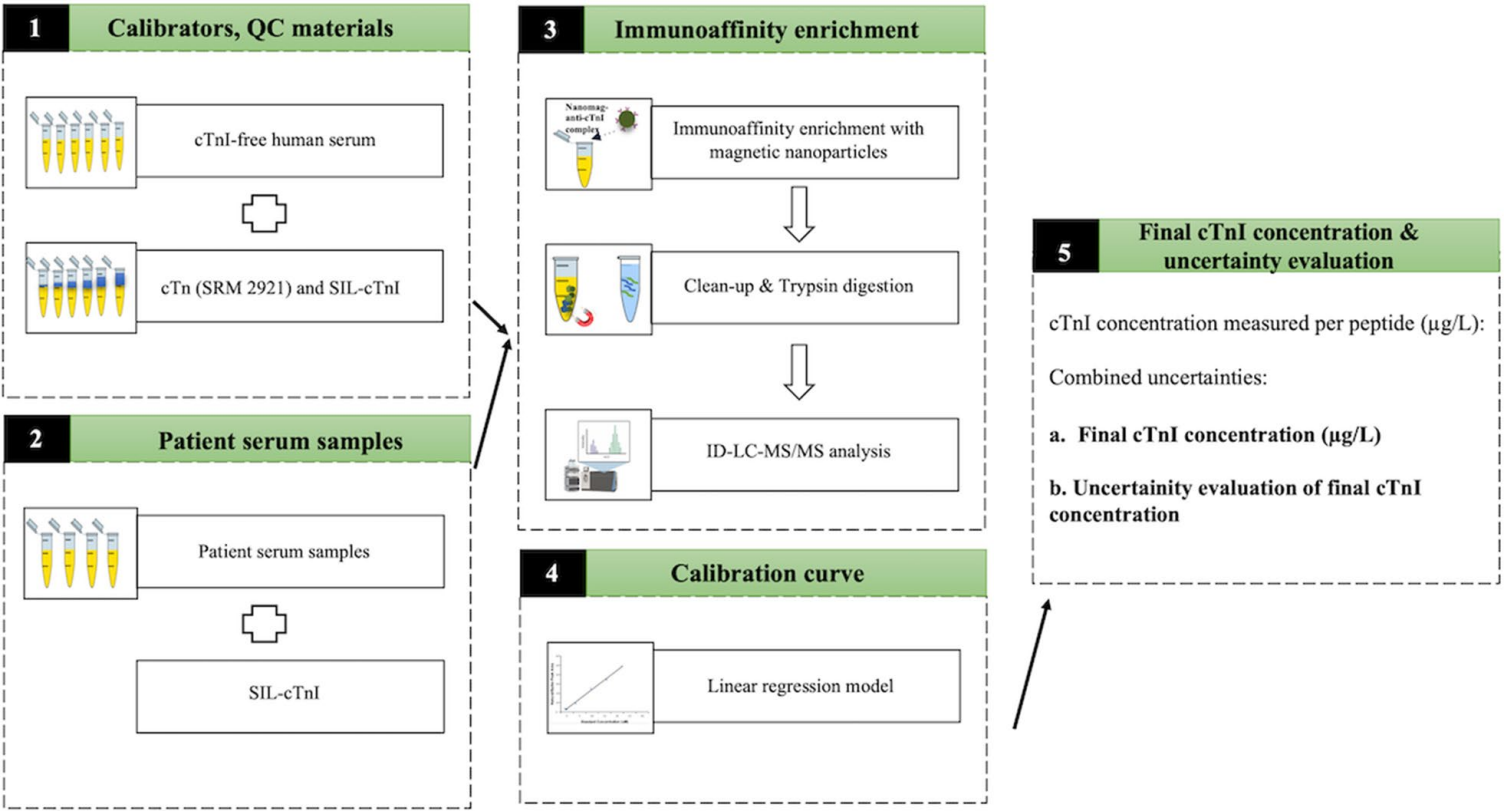
A diagrammatic illustrating the step-by-step of the analytical workflow for the SI-traceable quantification of cTnI in human serum. Step 1: the calibration standards and QC materials were prepared using cTnI-free serum by incorporating the NIST SRM 2921 and SIL-cTnI. Step 2: patient serum samples were prepared using cTnI positive serum samples by incorporating SIL-cTnI. Step 3: all samples were subjected immunoaffinity enrichment, followed by LC–MS/MS analysis. Step 4: calibration curves were established for the TLLLQIAK and NITEIADLTQK peptides to determine the concentration of cTnI. Step 5: to determine the uncertainty, all sources of uncertainty from steps 1 to 4 were combined

The sample preparation procedure for quantifying cTnI in serum is described in Fig. 1. Briefly, six-point calibration standards were prepared by spiking cTnI calibrator working solution into 900 µL cTnI-free human serum to obtain cTnI concentrations ranging from 0.7 to 24 µg/L. A constant volume of SIL-protein cTnI working solution was spiked to obtain a concentration of 15 µg/L. Similarly, three QC materials were prepared at concentrations of 2, 5, and 12 µg/L of cTnI, each containing 15 µg/L of SIL cTnI. Both calibrants and QCs were diluted with the same amount of PBS as the serum to reduce serum viscosity. Patient blood samples were collected in yellow capped biochemistry tubes from Kanuni Sultan Suleyman Hospital in Istanbul. Patient serum samples were obtained by spinning the blood at 2000×*g* for 10 min. The samples were obtained from individuals suspected of having a myocardial infarction. Immunoassay measurements of the samples were conducted immediately upon collection without freezing using the Siemens Atellica® Solution instrument. A reference range of cTnI with a threshold of 45 ng/L was utilized to distinguish between normal and elevated cTnI levels. Samples with cTnI concentrations exceeding 45 ng/L were categorized as “patient samples.” After collection, the serum samples were promptly frozen and stored at − 80 °C until the day of measurement using ID-MS. This ensured the preservation of sample integrity and minimized any potential degradation. Four patient serum samples were spiked with SIL cTnI to a final concentration of 15 µg/L. For immunoaffinity enrichment of cTnI, 10 µL anti-cTnI-magnetic bead complex was added to all calibrants, QCs and patient serum samples and incubated overnight at + 4 °C with continuous mixing. Non-specific proteins were removed by two washes with 20 mM Tris and 150 mM NaCl containing 0.05% Tween 20 followed by a single wash with ammonium bicarbonate in a magnetic separator for 30 min. All calibrants, QCs and patient serum samples were resuspended in 30 µL of 50 mM ammonium bicarbonate. Captured cTnI was digested using Abcam’s Filter-Aided Sample Preparation (FASP) according to the manufacturer’s instructions. Briefly, 10 mM DTT was used for 45 min at 60 °C to reduce disulfide bonds and alkylated with iodoacetamide (IAA) for 20 min in the dark at room temperature. Trypsin was added and digestion was carried out overnight at 37 °C. The digests were dried using SpeedVac. All samples were reconstituted with a final volume of 20 µL of SIL-DLPSPIER (0.18 ng/µL) to determine the relative amount of anti-cTnI antibody. The digested peptide mixtures were analyzed by Nano LC–MS/MS as described in Fig. 1.

### Bottom-up detection of cTnI enriched by magnetic beads

Analyses were done in PRM mode on the high-resolution mass spectrometry Thermo Scientific™ Mass Spectrometer Q-Exactive™ HF-X (Thermo Scientific, Waltham, MA, USA) coupled with Thermo Scientific™ Dionex™ Ultimate 3000 (Thermo Scientific) liquid phase chromatography [27]. The digested peptides were separated on an Acclaim™ PepMap™ 100 C18 analytical column (75 × 25 μm, 2 μm) (Thermo Scientific, Waltham, MA, USA). The following MS conditions were applied: capillary temperature at 270 °C, spray voltage of 2.0 kV, S-lens RF level of 50, sheath gas flow rate and auxiliary gas heater flow rate of 0. After optimization, 5 μL of the peptide digest was injected and the LC separation was carried out at a flow rate of 350 μL/min, a temperature of 40 °C using 100% water 0.1% of formic acid as mobile phase A and 20% acetonitrile in water, 0.1% of formic acid as mobile phase B. Peptides were eluted with the following gradient of mobile phase B: 3% for 3 min, linear from 24 to 36% in 5 min, linear from 36 to 80% in 7 min, constant in 80% for 8 min. MS2 scans were collected at a resolution of 15,000 with an automatic gain control target of 2e5, loop count of 8 and 30 microscan. Precursor ions were isolated within an isolation window a 0.6 m/z. Raw data were processed by Xcalibur Quan Browser software (Thermo Scientific). Peak areas were integrated using the Genesis algoritm. Signal extraction was applied within a mass tolerance of 15 ppm for PRM data. cTnI concentration was determined using calibration curves that were created using SRM 2921 as the calibrant. Calibration curves were plotted by correlating the peak area ratios of unlabeled to labeled cTnI peptide with the known ratios of the concentrations of unlabeled to labeled cTnI peptide.

### Method validation

The analytical performance for quantification of cTnI in human serum using SIL-cTnI as an internal standard was validated for linear range, accuracy, trueness, precision, and the presence of carryover [28]. The linearity of calibration curve was evaluated by regression analysis at cTnI concentrations of 0.7, 1.2, 3.5, 6, 12 and 24 μg/L. The peak area ratio of the analyte to the corresponding SIL-cTnI was plotted against the analyte concentration (μg/L). Correlation coefficients were determined for each curve. The linearity of the method was demonstrated by the recovery of the calibration samples. Recovery was reported as the percentage recovery of the measured concentration relative to the gravimetric concentration.

In addition, the accuracy of the quantification of each calibrant was evaluated by considering the calibrants as unknown samples. The individual concentration of each peptide was determined from its respective calibration curve. The trueness and precision were evaluated using three processed replicates of QC materials at three concentration levels (about 2.0, 5.0 and 12.0 µg/L) in four independent experiments using NIST SRM 2921. The mass ratio of each sample was obtained from calibration curve that was prepared on the same day. The coefficients of variation (CV) for intra-day and inter-day variations of the assay were determined. Intra-day CV was calculated by analyzing the samples at each concentration on the same day, whereas inter-day CV was determined by analyzing three samples at each concentration on 4 different days. The LOD and limit of quantification (LOQ) were determined by analyzing six replicate samples. For the carryover test, the signal observed at the retention time of each peptide (area below the peak) was less than 1% compared to that found in the LOD after injection of one blank sample.

The measurement uncertainty was determined according to the EURACHEM/CITAC Guide CG 4 (third edition) entitled “Quantifying Uncertainty in Analytical Measurement” [24, 25]. The uncertainty estimation considered various steps in the measurement process, including the uncertainty of the balance used for weighing, the uncertainty arising from the calibration curve, the assessment of intra-day and inter-day variances, and the evaluation of trueness. The derived overall uncertainty was multiplied by a coverage factor of k = 2, corresponding to an approximate confidence level of 95% assuming a normal distribution. This multiplication resulted in the calculation of the expanded uncertainty.

## Results

### Selection of proteotypic peptides

The first step in the development of a an PRM-MS based analysis is the selection of a subset of peptides to be used as quantitative representatives ‘Proteotypic peptides’ for each candidate protein [29, 30]. The identification of optimal proteotypic peptides representing the targeted proteins is very important for the accurate quantification of the target proteins using the targeted proteomics approach. ‘Proteotypic peptides’ must be sequence specific, detectable and the most responsive peptides. In this study, proteotypic peptides were selected using both computational and experimental strategies. The enhanced signature peptide predictor [29] was used to predict the high responding peptides for the cTnI protein. As a result of the analysis, seven peptides with the highest enhanced signature peptide predictor prediction factor were selected. These peptides are as follows, with the prediction factor from highest to lowest; NIDALSGMEGR, NITEIADLTQK, ISADAMMQALLGAR, TLLLQIAK, MADGSDAAR, EPRPAPAPIR and AYATEPHAK. The computational approach was also confirmed experimentally. Fully tryptic peptides of cTnI were screened using bottom-up proteomics. After trypsin digestion, the cTnI peptide solution was analyzed using with a Nano UPLC-Q-Exactive HF-X Orbitrap mass spectrometer with full MS/dd-MS2 (TopN) mode. Raw files were processed by Proteome Discoverer 2.5 (Thermo) using the Sequest algorithm. Advanced parameters of the Sequest algorithm were set as 10 ppm precursor mass tolerance, 0.02 Da fragment mass tolerance, static modifications of carbamidomethyl, dynamic modifications of acetyl and oxidation. Responses of tryptic cTnI peptides screened for PRM analysis are given in Table 1. Trypsin digestion hydrolyses the primary sequence of the target protein into specific peptide ending in C-terminal lysine or arginine residues. Both abundance and the number of peptide-spectrum match (PSMs) in the table aim to represent the response of the corresponding peptide, providing important insights into its relative abundance and confidence of identification. To assess potential phosphorylation sites of cTnI, we utilized the NetPhos-3.1 software, a generic phosphorylation site prediction tool for eukaryotic proteins. The results from NetPhos-3.1 indicated that threonine residues at positions 51, 124, and 129 were not predicted to be phosphorylated in cTnI. Based on this information, we selected two signature peptides, NITEIADLTQK and TLLLQIAK, for quantification. The primary selection criteria for these peptides were their high peptide-response and the fact that they have no known post-translational modifications, including phosphorylation [23, 31]. ISADAMMQALLGAR and NIDALSGMEGR gave high signal responses but were not selected due to their possible post-translational modifications. NITEIADLTQK and TLLLQIAK were chosen as surrogate peptides for the quantification of cTnI using ID-LC–MS/MS.

**Table 1:** Responses of tryptic cTnI peptides screened for PRM analysis and assay development

Sequence	Modifications	# PSMs	Positions	MH+ [Da]	Abundance
NITEIADLTQK	–	30	[121–131]	1245.6685	627,472,163
ISADAMMQALLGAR	2xOxidation [M6; M7]	22	[149–162]	1447.7395	583,636,382
NIDALSGMEGR	1xOxidation [M8]	23	[194–204]	1162.5521	544,496,892
TLLLQIAK	–	23	[51–58]	899.5924	499,353,997
AYATEPHAK	–	27	[28–36]	987.4894	195,415,852

### Immunoaffinity enrichment

In serum proteomics analysis, the presence of high-abundance proteins can significantly suppress the signal of low-abundance proteins, making it extremely difficult to analyze proteins with concentrations, such as cTnI in the low μg/L range [32]. In this study, immunoaffinity enrichment at the protein level was performed using a protein-based internal standard (NIST SRM 2921) to cope with the complexity and dynamic range of the serum to quantify cTnI by LC–MS/MS. This internal standard underwent the same sample processing as the endogenous cTnI, allowing us to assess potential problems such as incomplete protein extraction, incomplete proteolysis, or material loss during the sample preparation steps.

By using magnetic beads smaller than 1 μm in diameter, a higher capture efficiency can be achieved due to the higher surface area-to-volume ratio [13]. Therefore, the magnetic nanoparticles with particle diameters of 130 nm and the surface functionalities COOH for the covalent binding of the antibody was selected for immobilization of anti-cTnI antibody.

**Fig. 2:**
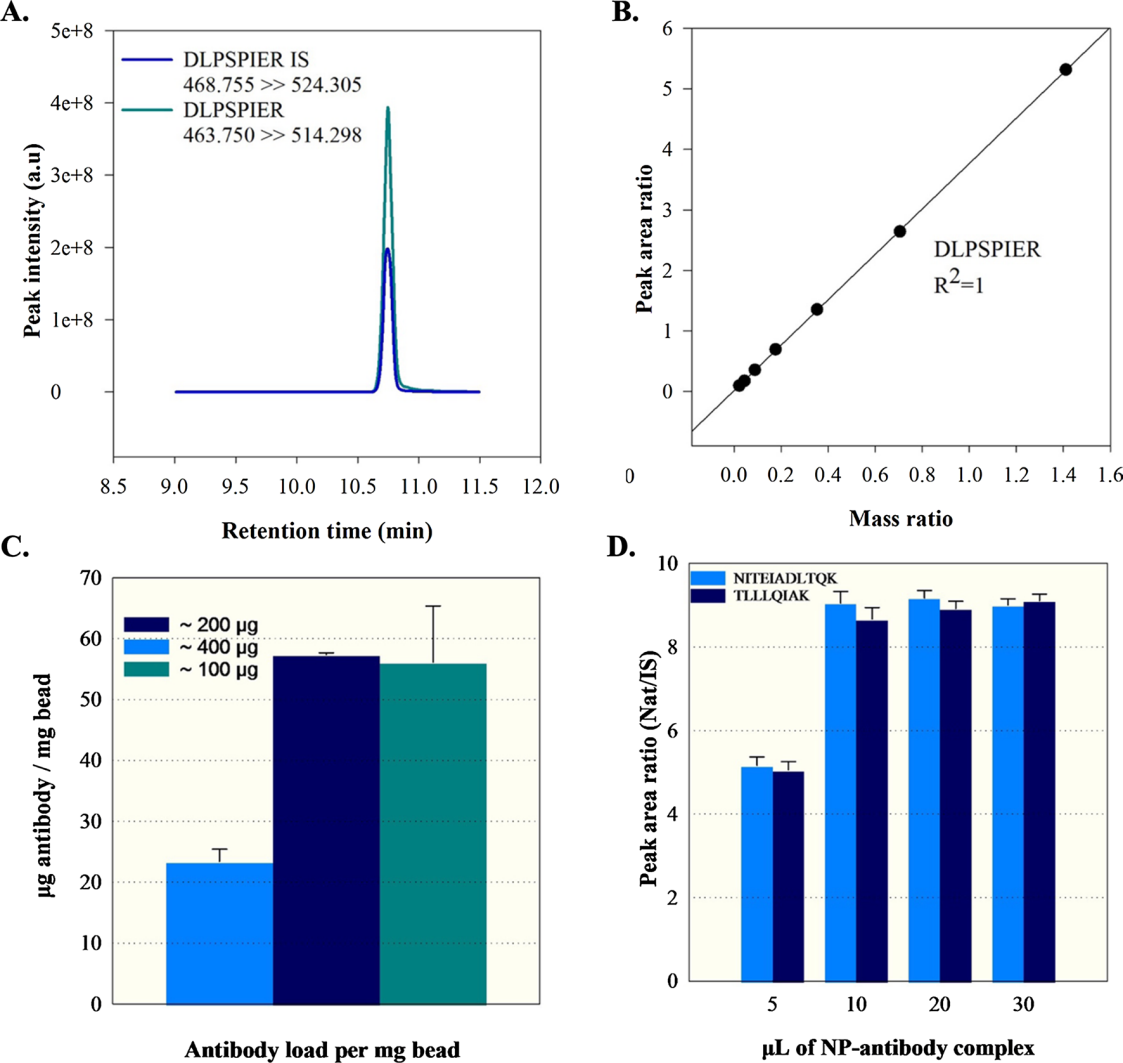
**A** Extracted ion chromatograms (EIC) for both DLSPIER and IS-DLSPIER peptides. **B** The Calibration Curve, **C** bar graph depicting the amount of immobilized anti-cTnI antibody per milligram of nanoparticles. The standard deviation error bars indicate the variability between duplicate preparations of the conjugates. **D** Bar graph depicting the amount of NP-antibody conjugate required for 1 mL serum enrichment

The ID-LC–MS/MS method presented by Schneck et al. was used to directly quantify and measure the amount of antibody bound to magnetic nanoparticles [13]. Figure 2A–D illustrates the methodology used to quantifying the antibody bound to magnetic particles using the ID-LC–MS/MS technique. Briefly, matrix-matched external calibrants were prepared by combining the antibody and internal standard in a manner that mimicked the sample matrix. The heavy isotope labeled synthetic DLPSPIER peptides were purchased as internal standards because of their uniqueness to the constant region of the monoclonal antibody (mAb), robust MS/MS signal intensity and absence of amino acid residues prone to chemical modification, such as cysteine or methionine residues. These calibrants were used to establish a calibration curve for accurate quantification. Following antibody immobilization, the antibody-magnetic particle conjugates were extensively washed to remove non-specifically adsorbed antibodies. The immobilized antibodies were then subjected to in situ trypsin digestion together with the magnetic particles. Internal standard was added to the digested samples, which were then analyzed directly by LC–MS/MS. Quantification was performed by monitoring a specific transition for DLSPIER peptides. To determine the maximum loading capacity of the magnetic nanoparticles, different antibody concentrations were used while keeping the amount of nanoparticles constant. In this study, approximately 100, 200, and 400 μg of antibody were added to the immobilization solutions containing 1 mg of beads. It was observed that the maximum surface loading of antibodies onto the magnetic nanoparticles was achieved at a ratio of approximately 100 μg antibody per mg of nanoparticles. In addition, further increases in antibody loading resulted in a decreased in efficiency. When immobilizing of antibodies on the surface of nanoparticles by physical adsorption, it is crucial to select the optimal antibody concentration for each specific case, which is not necessarily the highest concentration available [33]. This suggests that at high antibody concentrations, a portion of the nanoparticle surface that is not accessible for antibody binding due to steric hindrance. As shown in Fig. 2C, the highest amount of immobilized antibody was achieved by conjugating 1 mg of nanoparticles with 100 μg of antibody (59.2 ± 5.7 μg/mg). To calculate the amount required for cTnI enrichment from 1 mL of serum using the synthesized nanoparticle (NP)-antibody conjugate, enrichment was performed using 5, 10, 20, and 30 μL of NP-antibody conjugate. As shown in Fig. 2, the amount of peptide measured reaches saturation after the use of 10 μL of NP-antibody conjugate in serum cTnI enrichment. Therefore, the use of 10 μL of conjugate appears to be sufficient to capture all of the cTnI in 1 mL of serum for the analysis. The remaining part of the study was performed using these optimized parameters.

### Quantification of cTnI in human serum

Monitoring more than one proteotypic peptide as a quantifier increases confidence in the accuracy of protein analysis in matrix-based assays. Considering the wide range of cTnI modifications and the variability of cTnI isoforms among different patients’ plasma, the measurement of multiple peptides provides greater confidence and accuracy [34]. Therefore, the proteotypic peptides NITEIADLTQK and TLLLQIAK were selected for cTnI quantification and simultaneously monitored by PRM. The collision energies for each peptide were optimized experimentally optimized by PRM monitoring. The optimized parameters were utilized to observe the fragmentation transitions of the two cTnI peptides (NITEIADLTQK and TLLLQIAK) and a single antibody peptide (DLPSPIER) in both labeled and unlabeled forms. Table 2 summarizes the PRM transitions of selected peptides for cTnI measurement by the nanoparticle enrichment method using the intact protein internal standard (NIST SRM 2921).

**Table 2:** PRM transitions, amino acid sequence and retention time for the detection of the peptides

Proteotypic peptides	Retention time (min)	Precursor ions (m/z)	Collision energy (eV)	Product ions (m/z)
TLLLQIAK	13.89	450.30	20	572.377
				685.460
TLLLQIAK (U-N15)	13.89	455.28	20	579.356
				693.437
NITEIADLTQK	11.28	623.35	20	675.367
				1018.542
NITEIADLTQK (U-N15)	11.28	630.31	20	683.311
				1029.506
DLPSPIER	9.73	463.750	25	514.298
				698.383
DLPSPIER (13C6, 15N4)	9.73	468.755	25	524.305
				708.390

**Fig. 3:**
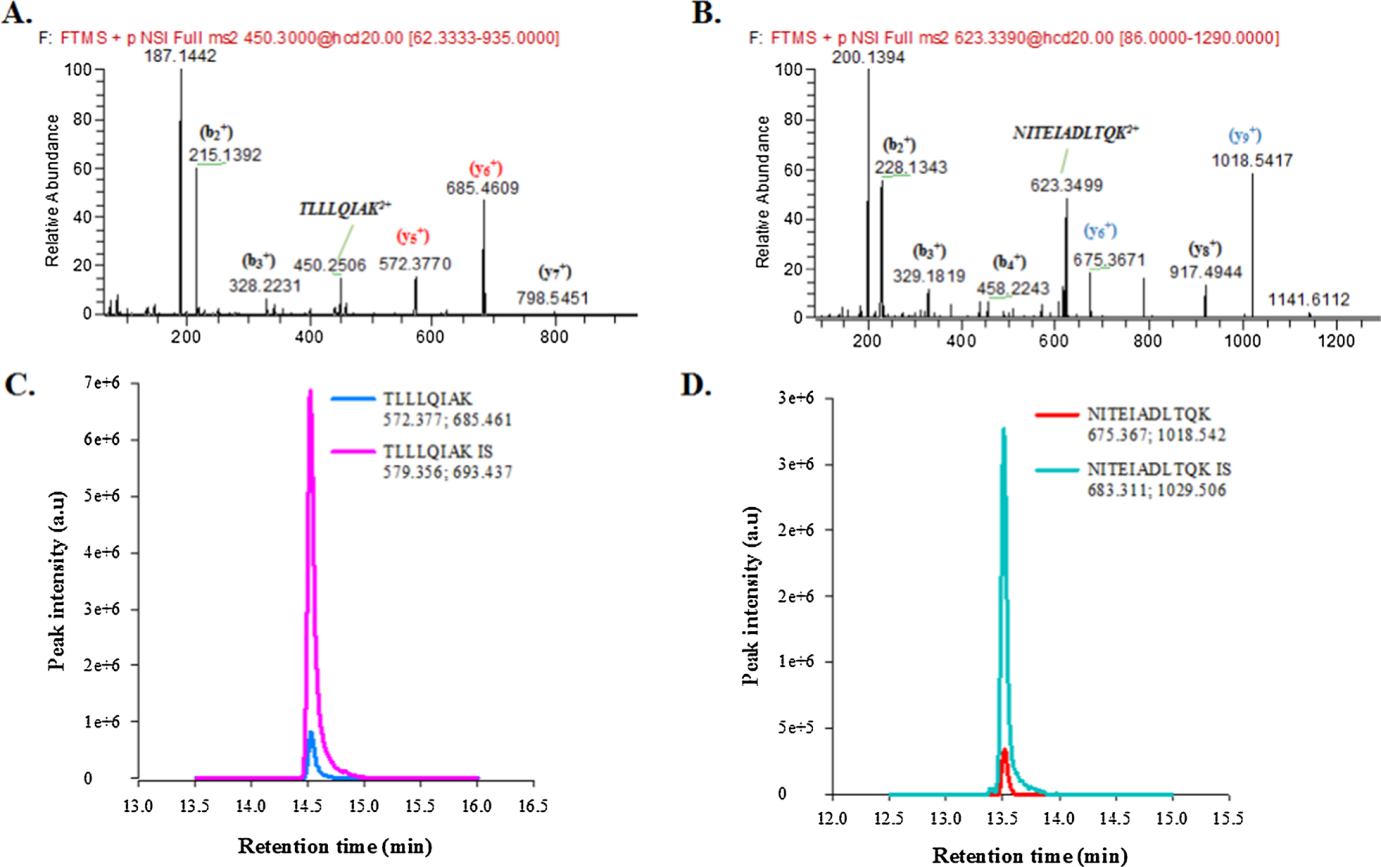
PRM analysis of tryptic digests of cTnI spiked serum. **A** MS/MS spectrum of targeted precursor ion TLLLQIAK^2+^ (selected product ions y_5_^+^ and y_6_^+^ for quantification in red); **B** MS/MS spectrum of targeted precursor ion NITEIADLTQK^2+^ (selected product ions y_6_^+^ and y_9_^+^ for quantification in blue); **C** EICs obtained when quantifying cTnI-free human serum spiked with a cTnI at LOQ level (1.8 µg/L) and SIL-cTnI at 15 µg/L showing coelution of the TLLLQIAK peptide and its isotopically labeled counterpart; **D** EICs obtained when quantifying cTnI-free human serum spiked with a cTnI at LOQ level (1.8 µg/L) and SIL-cTnI at 15 µg/L showing coelution of the NITEIADLTQK peptide and its isotopically labeled counterpart. Precursor ions were isolated using an isolation window of 0.6 m/z

The PRM MS/MS spectrum of the selected cTnI proteolytic peptides and the corresponding extracted ion chromatograms are shown in Fig. 3. The peak areas of the selected proteotypic peptides were used to establish a calibration curve based on IDMS and quantitative analysis.

This study utilized NIST SRM 2921, a protein calibrant, along with its labeled internal standard, to establish a calibration curve. Endogenous cTnI from patient serum samples and SIL-cTnI were isolated by immunoaffinity enrichment strategy and subsequently analyzed by ID-MS. There are different forms of cTnI in blood such as triple complex (cTnC-cTnT-cTnI), binary complex (cTnI-cTnC), free form, proteolyzed form, heparin bound form, phosphorylated form, oxidized and reduced forms. Furthermore, cTnI can be degraded both in vivo and in vitro [35]. Due to this variability and heterogeneity, there is no perfect matrix-matched protein calibrant for LC–MS quantification strategy. Nevertheless, SRM 2921 has been purified from human heart tissue and because of its heterogeneity is currently the best choice for purpose [26].

### Method validation

#### Linear range

The method was validated using the NIST SRM 2921 as calibrant. To assess the linearity of the method response, the integrated peak area ratios of cTnI peptide transitions (unlabeled to labeled) were plotted against the mass ratios of SRM 2921. The quantities used for the plot ranged from 0.7 to 24 μg/L, with specific concentrations of 0.7, 1.2, 3.5, 6, 12 and 24 μg/L. The peak areas were automatically integrated by computer using the Quan Browser software program (Thermo Scientific). The method response was found to be linear for between 0.7 μg/L and 23.3 μg/L of cTnI with a regression coefficient of 0.996 and 0.992 for the peptides TLLLQIAK and NITEIADLTQK respectively according to the equations presented in Table 3. The LOD was calculated as defined by ICH [28] using the formula LOD = 3 × Sa/b, where b is the slope of the calibration curve and Sa is the standard deviation of the intercept. The LOD values were found to be 0.6 ng and 1.6 ng for cTnI using the peptides TLLLQIAK and NITEIADLTQK for quantification, respectively. The LOQ was also determined according to the defined criteria, LOQ = 10 × Sa/b [28], and was found to be 1.8 ng and 4.8 ng for cTnI using the peptides TLLLQIAK and NITEIADLTQK for quantification, respectively.

**Table 3:** Linear range of method response by surrogate peptides TLLLQIAK and NITEIADLTQK

Parameters	TLLLQIAK	NITEIADLTQK
Optimum range for cTnI (μg/L)	0.7–23.3	0.7–23.3
Regression equation	Y_ratio peak area_ = 2.6219C_cTnI_-2.3795	Y_ratio peak area_ = 1.7415C_cTnI_ − 1.2778
Correlation Coefficient	0.996	0.992
LOQ of cTnI (μg/L)	1.8	4.8
LOD of cTnI (μg/L)	0.6	1.6

#### Accuracy

The accuracy is defined under precision and trueness of the test results. For precision of the measurement system, the repeatability and intermediate precision of the method were evaluated by intra-day (analysis of QC solutions in replicates of three in the same day) and inter-day (in replicates of three on four different days) assay variance, respectively. At the QC concentration levels evaluated, the repeatability (intra-run) and intermediate precision (inter-run) of the acceptance standards should be less than 10% (percent RSD). RSD_Repeatability_ (1) and RSD_intermediate precision_ (2) were calculated using the formulas given below. For QCs, the values obtained for repeatability and intermediate precision were less than 10% RSD (Table 4). These values met the acceptance requirements, indicating that the current method has sufficient precision.

1 $$RSD_{Repeatability} = \frac{{\sqrt {MS_{within} } }}{{W_{mean} }} \times 100,$$

2 $$RSD_{IntermediatePrecision} = \frac{{\sqrt {MS_{between} - MS_{within} } }}{{W_{mean} }} \times 100.$$

**Table 4:** The precision results of different cTnI concentrations

	TLLLQIAK (μg/L)			NITEIADLTQK (μg/L)		
	2	5	12	2	5	12
**Repeatability (n = 3)**						
Mean ± SD	2.1 ± 0.1	4.7 ± 0.8	11.9 ± 0.1	2.0 ± 0.1	4.9 ± 0.3	13.3 ± 0.3
RSD_Repeatability_	4.9	6.8	2.2	8.7	4.4	2.0
**Intermediate precision (n = 12)**						
Mean ± SD	2.1 ± 0.1	4.9 ± 0.4	11.4 ± 0.5	2.1 ± 0.2	5.0 ± 0.5	12.7 ± 0.7
RSD_Int.precision_	6.4	9.6	5.2	8.7	4.4	7.6

The trueness assessment was performed with the recovery evaluated using NIST SRM 2921. Three QC levels 2.0 (low), 5.0 (medium), and 10.0 (high) μg/L were assessed for each peptide. The obtained recovery data for TLLLQIAK was 104.2%, 97.3% and 94.8% for 2, 5 and 10 μg/L cTnI levels respectively. The obtained recovery data for NITEIADLTQK was 104.5%, 99.9% and 106.1% for 2, 5 and 10 μg/L cTnI levels respectively. Accordingly, % recovery values were calculated and presented in Table 5. Those values met the recovery acceptability requirements, indicating that the current approach was adequate in terms of trueness.

**Table 5:** The trueness results of different cTnI concentrations

% Trueness (n = 3)	TLLQIAK (μg/L)			NITEIADLTQK (μg/L)		
	2	5	12	2	5	12
Mean, Day1	106.9	94.1	99.4	101.7	98.2	110.8
Mean, Day2	96.6	101.4	92.5	100.7	103.1	108.2
Mean, Day3	107.2	89.6	96.3	111.2	100.8	107.8
Mean, Day4	105.9	104.0	91.0	104.4	97.6	97.5
Mean ± SD	104.2 ± 4.4	97.3 ± 5.7	94.8 ± 3.3	104.5 ± 4.1	99.9 ± 2.2	106.1 ± 5.1

#### Carryover

In the present method, a solution of acetonitrile/water (80/20; v/v) with 0.1% formic acid was used to rinse the syringe and injection port. This wash procedure was performed several times before and after each injection. Under these washing conditions, the signal observed at the retention time of each peptide (area below the peak) was less than 1% compared to that found in the LOD after injection of a blank sample.

### Evaluation of measurement uncertainty

The uncertainty of the method was evaluated according to EURACHEM/CITAC Guide CG 4 (third edition) entitled “Quantifying Uncertainty in Analytical Measurement” [24, 25]. Uncertainty sources of arising from operations steps such as pipetting, dilution, balances and volumetric equipment are covered by the reproducibility, intermediate precision and recovery uncertainties. The sources of uncertainty identified for the present method are the following defined parameters: balance, the repeatability standard deviation s_r_ (3), the contribution of the grouping factor to the total variation (s_between_) (4) the uncertainty of the calibration curve (5). The formula of the standard combined uncertainty (6) given below. The expanded uncertainty has been calculated considering a coverage factor of 2 for a confidence of approximately 95%. The breakdown of the uncertainty budget is presented in Table 6.

3 $$s_{r} = \sqrt {MS_{withingroup} } ,$$

4 $$s_{between} = \sqrt {\frac{{MS_{within} - MS_{between} }}{{n_{repeat} }}} ,$$

5 $$u_{cal} = \frac{s}{m} \times \sqrt {\frac{1}{{n_{rep} }} + \frac{1}{{n_{cal} }} + \frac{{\left( {x_{pred} - x_{mean} } \right)^{2} }}{{\sum \left( {x_{i} - x_{mean} } \right)^{2} }}} ,$$

6 $$u_{combined} = k\sqrt {\frac{{u_{rep}}^{2}}{n_{rep}} + \frac{{u_{intPre}}^{2}}{n_{days}} + {u_{calibration}}^{2} + {u_{balance}}^{2}} .$$

**Table 6:** Breakdown of the uncertainty budget

	TLLQIAK (μg/L)			NITEIADLTQK (μg/L)		
	2	5	12	2	5	12
Mean	2.1	4.9	11.4	2.1	5.0	12.7
**Uncertainty components**						
s_r_	0.1	0.2	0.3	0.2	0.2	0.5
s_between_	0.1	0.3	0.2	0.2	0.2	0.3
u_cal_	0.2	0.2	0.2	0.2	0.2	0.2
Balance	0.0	0.0	0.0	0.0	0.0	0.0
k	2	2	2	2	2	2
Uncertainty, u_c_	0.3	0.5	0.5	0.3	0.4	0.6
Expanded uncertainty, U	0.6	0.9	0.9	0.7	0.7	1.2

### Application to clinical samples

To evaluate the results of the developed reference method, the candidate reference method was applied to patient serum samples. Although we have almost mimicked the patient sample by adding the human cTn complex to human cTnI free serum, it is still important to quantify the developed method on clinical samples considering variety of cTnI modification and unique cTn profile of individuals. Unfortunately, in all samples collected (n = 25), the levels of cTnI were found to be below the LOQ of our method. From all the collected patient samples, the four samples which cTnI values are above the detection limit were selected and analyzed by the developed ID-LC–MS/MS method.

Table 7 presents the results obtained from both the ID-LC–MS/MS method and the Siemens Atellica® Solution immunoassay. The recovery and uncertainty was not calculated because the cTnI values of the selected samples, as detected by the immunoassay, were below the quantification limit of the developed ID-MS method. Based on the results of the validation study, we predicted that these samples could be detected but might not provide fully accurate quantitative results. The experimental results confirmed our prediction. However, the fact that the developed methodology performed similarly on the patient samples demonstrates the reliability of the method. This is further supported by the high recovery values observed for the QC samples prepared at concentrations of 2, 5, and 12 µg/L, as shown in Table 5.

**Table 7:** Application to clinical samples

	cTnI concentration from the Siemens Atellica® Solution Immunoassay measurements (µg/L)	ID-LC–MS/MS	
		TLLLQIAK (µg/L)	NITEIADLTQK (µg/L)
Patient1	0.6	0.4	0.5
Patient2	1.6	0.8	0.8
Patient3	0.7	0.6	0.5
Patient4	0.6	0.3	0.4

In future developments, it is important to improve the developed method by achieving a lower LOQ. Additionally, a larger number of samples should be utilized to assess the applicability of the method to clinical samples.

## Discussion

Two different ID-LC–MS/MS methods were used in this study. The first method was used to quantify anti-cTnI antibody bound to magnetic beads, while the second method was used to quantify cTnI in human serum. These two methods allowed for the specific and accurate measurement of both the antibody and the target protein in their respective matrices.

ID-LC–MS/MS offers advantages such as the ability to differentiate interfering substances, independence from specific reagents, and direct measurement of targeted peptides, making it a valuable method for quantifying antibodies immobilized to magnetic particles and assessing the quality of the enrichment process. The efficiency of the enrichment is undoubtedly influenced by a number of factors, including the physiochemical characteristics of the antibody/magnetic particles, the method used to immobilize the antibody, and the surface properties of the magnetic particle. Together, these factors contribute to the overall performance and effectiveness of the enrichment process. An essential factor for successful protein capture is the presence of an adequate amount of antibody bound to the surface of the magnetic particles. In this study, magnetic nanoparticles were chosen for protein enrichment because of their superior ability to bind antibodies compared to microparticles. In a previous study, four types of magnetic particles were used to conjugate anti-cTnI antibody. These particles included both microparticles and nanoparticles, which were coated with epoxy and amine groups on their surfaces [13]. According to the study, the researchers observed that the nanoparticles coated with glutaraldehyde groups yielded the highest concentration of immobilized antibody. Specifically, by adding approximately 300 μg of antibody, they were able to achieve an antibody concentration of 44 μg/mg of magnetic particles. This finding suggests that the nanoparticles with glutaraldehyde groups were the most effective in facilitating the conjugation and immobilization of the antibody onto the magnetic particles. In this study, to further investigate the impact of various characteristics of magnetic particles on their enrichment performance we conduct our experiments using commercially available magnetic nanoparticles that were coated with COOH groups and capable of covalently binding to antibodies. Our results showed a significant improvement in the immobilization of anti-cTnI, with more than two and a half times better immobilization at a rate of 59.2 ± 5.7 μg/mg. Interestingly, this improved immobilization was achieved by using a lower amount of antibody, specifically 100 μg. Consequently, we were able to immobilize a higher amount of antibodies onto the magnetic particles while minimizing the amount of antibody required. These observations highlighted the importance of optimizing the antibody-to-nanoparticle ratio to achieve the desired immobilization efficiency. By determining the optimal ratio, researchers can ensure the maximum utilization of both the nanoparticles and the antibodies, leading to improved performance in various applications that rely on antibody immobilization, such as biosensors, immunoassays, or magnetic separation techniques.

As noted in the previous section, Schneck et al. [23] have previously developed an ID-LC–MS/MS method for the quantification of cTnI in plasma. The reported cTnI values ranged from 4.9 to 11.3 µg/L. Although the study provides validation parameters, it lacks information on the LOD, LOQ, and uncertainty budget specifically for the plasma or serum samples. The LOD and LOQ values reported in the study, 163 pg and 325 pg respectively, are given for measurements in buffer rather than plasma or serum. The LOD and LOQ values in buffer may not accurately reflect the limit of detection and quantification achievable in plasma or serum due to potential matrix effects and interferences. Furthermore, uncertainty estimation is crucial for assessing the reliability and accuracy of measurement results, taking into account various sources of uncertainty in the analytical process.

The method developed and validated in our study for the quantification of cTnI in human serum has LOQ of 0.6 µg/L and LOD of 1.8 µg/L. These values indicate the lowest concentration of cTnI that can be reliably quantified or detected in human serum samples using an ID-LC–MS/MS method. Furthermore, in this study, a recovery rate of approximately 95% was achieved using only 10 μL of antibody-nanoparticle conjugates for the enrichment of cTnI from human serum. This indicates that the method efficiently captures and recovers the target analyte from the serum samples, demonstrating its effectiveness in sample preparation and enrichment for cTnI analysis.

Furthermore, in this study, a recovery rate of approximately 95% was achieved using only 10 μL of antibody-nanoparticle conjugates for the enrichment of cTnI from human serum. This indicates that the method effectively captures and recovers the target analyte from the serum samples, demonstrating its efficiency in sample preparation and enrichment for cTnI analysis.

Previous studies have reported that approximately 30% of cTnI was recovered using small amounts of SRM 2921 were used from human plasma [23, 36]. Furthermore, Schneck et al. reported a recovery of cTnI with 93% efficiency using 30 μL of antibody-nanoparticle conjugates, but it is important to note that this recovery was observed in a buffer solution (specifically, 0.2% BSA in PBS, 1.8 mL) rather than in human serum [36]. The higher recovery rate observed in this study with human serum samples using a smaller volume of antibody-nanoparticle conjugates suggests the effectiveness of the developed method for cTnI enrichment in serum samples.

In contrast to the commonly used quantitative MS-based assays, the method developed in this study was integrated into a Nano LC system. The aim was to establish a reliable and reproducible method for measurement of cTnI in human serum. After processing, the protein was recovered within a concentration range of 0.7–24 μg/L. It is noteworthy that the LOQ and LOD values obtained in this study were lower compared to previous studies that measured cTnI using MS-based methods. This suggests that the method developed in this study has improved sensitivity in detecting lower concentrations of cTnI in human serum samples [15, 17, 19, 23]. Furthermore, the correlation coefficient (r) of the calibration curve was found to be greater than 0.996. This high correlation coefficient indicates a strong and reliable relationship between the concentration of cTnI in the serum samples and the corresponding signal response obtained from the peptides used for detection. The high correlation coefficient of the calibration curve confirms the accuracy and precision of the developed method for the quantification of cTnI in serum.

Immunoassays for troponin proteins are commonly used to accurately measure their concentrations in biological samples. These assays are capable of detecting and quantifying troponin proteins in the low picogram/milliliter range [5]. MS-based assays may not achieve the same level of sensitivity as the most sensitive clinical assays, but they can offer other advantages such as specificity and the ability to provide accurate measurements of target analytes. By developing a candidate reference method using MS-based techniques our aim was to establish a method that could be used for the comparability of commercial immunoassays for cTnI at clinically relevant concentrations.

The development of SI-traceable measurement methods for the quantification of biomarkers, such as cardiac troponin I (cTnI), represents a significant advancement in ensuring the accuracy and reliability of measurement outcomes. By establishing a measurement method that is directly linked to international standards, the process of quantifying cTnI can be standardized across various laboratories and manufacturers. It is noteworthy to address the discontinuation of the NIST SRM 2921 in this context. While the CRM’s discontinuation is acknowledged, the validity of its certificate ensures the ongoing availability of a steadfast reference point for our study. Importantly, this situation does not compromise the fundamental fact that our developed method remains rigorously traceable to the SI unit. As a result, the integrity and reliability of our method’s traceability to international standards remain unwavering, providing a solid foundation for accurate cTnI quantification. The adoption of such standardized approaches significantly reduces the likelihood of inaccurate diagnoses, minimizing the potential repercussions of false-positive or false-negative results. This proactive approach contributes to the overall reliability and effectiveness of medical diagnostics, benefiting patients, healthcare providers, and the medical community at large.

## Conclusions

This paper presents a validated measurement protocol that utilizes the ID-LC–MS/MS method. While we initially aimed to create a comprehensive RMP, we openly recognize that the current limit of quantification (LOQ) presents a challenge in detecting lower cTnI concentrations. Our developed method demonstrates significant strengths while also offering potential for further improvement. The presented protocol allows for the absolute quantification of cTnI in human, ensuring SI- traceability and covering a wide range of clinical cut-off concentrations. By incorporating the use of NIST SRM 2921 as a primary standard, traceability to the SI-unit has been established. Incorporating a primary calibrator and internal standard, the accuracy of the method greatly improved and challenges associated with incomplete digestion and material loss during sample preparation were effectively addressed.

The developed method has the potential to serve as a RMP for the determination of cTnI concentration in external quality assessment materials and secondary CRMs. This would allow for the comparison of results obtained from commercial immunoassays for cTnI at clinically relevant concentrations, ensuring their comparability. The proposed measurement method will play a crucial role in supporting the activities of IFCC WG-TNI (International Federation of Clinical Chemistry and Laboratory Medicine Working Group on Cardiac Troponin) and addressing the standardization of cTnI assays. This is a necessary and logical step towards the harmonization of the results obtained from different test kits.

## Data Availability

Data are available from the corresponding author upon reasonable request.

## Abbreviations

CRM: Certified reference materials
cTn: Cardiac troponin
cTnI: Cardiac troponin I
DTT: Dithiothreitol
EDC: 1-Ethyl-3-(-3-dimethylaminopropyl) carbodiimide hydrochloride
FASP: Filter-aided sample preparation
IAA: Iodoacetamide
IDMS: Isotope dilution mass spectrometry
ID-LC–MS/MS: Isotope dilution liquid chromatography–tandem mass spectrometry
IFCC WG-TNI: International Federation of Clinical Chemistry and Laboratory Medicine
LC–MS: Liquid chromatography mass spectrometry
LOD: Limit of detection
LOQ: Limit of quantification
MES: 4-Morpholineethanesulfonic acid
MS: Mass spectrometry
NHS: *N*-Hydroxysuccinimide
NIST: National Institute of Standards and Technology
NP: Nanoparticle
PBS: Phosphate-buffered saline
PRM: Parallel reaction monitoring
QC: Quality control
RMP: Reference measurement procedure
RSD: Relative standard deviation
SI: International System of Units
SIL: Stable isotope labeled
SRM: Standard Reference Material
